# Hospitalization is a missed opportunity for HIV screening, pre-exposure prophylaxis, and treatment

**DOI:** 10.1186/s13722-024-00451-z

**Published:** 2024-03-26

**Authors:** William Bradford, Hana Akselrod, John Bassler, Kelly W. Gagnon, Greer Burkholder, Joseph Edward Carpenter, Alaina Steck, Jillian Catalanotti, Irene Kuo, Keanan McGonigle, William Mai, Melissa Notis, Christopher Brokus, Sarah Kattakuzhy, Elana Rosenthal, Ellen F. Eaton

**Affiliations:** 1 Division of Infectious Diseases, University of Alabama at Birmingham, Boshell Building 8th Floor 1808 7th Ave S, Birmingham, AL 35233 USA; 2 Department of Medicine, The George Washington School of Medicine and Health Sciences, Washington, USA; 3 School of Public Health, University of Alabama at Birmingham, Birmingham, USA; 4 Department of Emergency Medicine, Emory University School of Medicine, Atlanta, USA; 5 Department of Epidemiology, Milken Institute School of Public Health, The George Washington University, Washington, USA; 6 Division of Clinical Care and Research, Institute of Human Virology, University of Maryland School of Medicine, Baltimore, USA; 7 Division of Infectious Diseases, University of Alabama at Birmingham, Boshell Building 8th Floor 1808 7th Ave S, Birmingham, AL 35233 USA

**Keywords:** OUD, HIV, PrEP, PWH, Missed opportunity, SIRI, CHOICE

## Abstract

**Background:**

Hospitalization is a “reachable moment” for people who inject drugs (PWID), but preventive care including HIV testing, prevention and treatment is rarely offered within inpatient settings.

**Methods:**

We conducted a multisite, retrospective cohort study of patients with opioid use disorder with infectious complications of injection drug use hospitalized between 1/1/2018–12/31/2018. We evaluated HIV care continuum outcomes using descriptive statistics and hypothesis tests for intergroup differences.

**Results:**

322 patients were included. Of 300 patients without known HIV, only 2 had a documented discussion of PrEP, while only 1 was prescribed PrEP on discharge. Among the 22 people with HIV (PWH), only 13 (59%) had a viral load collected during admission of whom all were viremic and 10 (45%) were successfully linked to care post-discharge. Rates of readmission, Medicaid or uninsured status, and unstable housing were high in both groups.

**Discussion:**

We observed poor provision of HIV testing, PrEP and other HIV services for hospitalized PWID across multiple U.S. medical centers. Future initiatives should focus on providing this group with comprehensive HIV testing and treatment services through a status neutral approach.

## Background

Rates of inpatient admission for people who inject drugs (PWID) are increasing [1], coinciding with a rise in serious injection-related infection, overdose deaths, and HIV acquisition [2]. Pre-exposure prophylaxis (PrEP) awareness and uptake among PWID and providers remain poor [3] despite high HIV risk, more than a decade of experience with FDA-approved PrEP regimens, and a renewed national focus on reducing HIV rates through initiatives such as the Ending the HIV Epidemic [2]. Hospitalization is considered a “reachable moment” for PWID for a variety of interventions [4], but data are limited on the provision of HIV testing, PrEP and HIV treatment for hospitalized PWID. We sought to evaluate the HIV continuum of care among individuals hospitalized with infectious complications of injection opioid use.

## Methods

The Continuum of Care in Hospitalized Patients with Opioid Use Disorder and Infectious Complications of Drug Use (CHOICE) study is a retrospective cohort study of patients with opioid use disorder (OUD) hospitalized with infectious complications of injection opioid use performed at four sites across the Southeast and Mid-Atlantic United States [5]. CHOICE included patients hospitalized between 1/1/2018–12/31/2018 with ICD10 diagnosis codes consistent with both OUD (mainly F11 series ICD-10 codes) and acute bacterial/fungal infection as well as chart verification of injection-associated infection at sentinel admission. Data were abstracted from electronic medical records for a 12 month follow up period starting at the date of hospital admission, including data on HIV status, labs, consultations, medications, and rates of linkage to outpatient care within each institution in the year following discharge from sentinel admission. For this analysis, we focused specifically on HIV treatment among persons living with HIV (PWH) and PrEP prescription for patients living without HIV. We calculated descriptive statistics and summarized demographic and clinical factors using measures of central tendency (sample medians), dispersion (interquartile range), and distribution (frequency, percentage). Statistical assumptions and sample size requirements were assessed prior to hypothesis testing, and when appropriate, formal testing of factors by HIV status (Kruskal–Wallis, Pearson Chi-Square test; Fisher’s Exact Test) were evaluated at the p < 0.05 significance level. Missing data were considered missing at random and, therefore, were not included in descriptive measures or statistical tests. All analyses were conducted using SAS software (v.9.4) of the SAS System for Windows.

## Results

Overall, 322 patients met inclusion criteria for the CHOICE study (Table 1) [5]. Skin and soft tissue infection (SSTI) was the most common injection-related infection (64.9%), followed by bacteremia (34.2%), endocarditis (15.8%), and osteomyelitis (14.9%). Septic arthritis (4.3%) and epidural abscess (5.9%) were less common. The median age was 38 years and median hospital length of stay was six days.

**Table 1:** Demographic and clinical characteristics of patients according to HIV status

	People without known HIV (N = 300)	People with HIV (N = 22)	P-value
Patient age (years)	38 (31, 50)	48 (34, 57)	**0.02** ^1^
Length of stay (days)	6 (3, 15)	10 (5, 31)	**0.02** ^1^
Sex (Male)	176 (59%)	15 (68%)	0.38 ^2^
Race			
White	203 (68%)	10 (45%)	0.06 ^3^
Black	89 (30%)	12 (55%)	
Other	8 (3%)	0	
Insurance status			
Medicaid	141 (47%)	17 (77%)	0.20 ^3^
Uninsured	94 (31%)	4 (18%)	
Medicare	23 (8%)	0 (0%)	
Not documented	15 (5%)	1 (5%)	
Other	15 (5%)	0 (0%)	
Commercial	12 (4%)	0 (0%)	
Housing status			
Stable	146 (49%)	10 (46%)	0.51 ^2^
Unstable	105 (35%)	10 (46%)	
Unknown	49 (16%)	2 (9%)	
Discharge status			
Routine	231 (77%)	18 (82%)	0.999 ^3^
PDD	60 (20%)	4 (18%)	
Death	5 (2%)	0	
Other	4 (1%)	0	
Infection causing hospitalization			
SSTI	197 (66%)	12 (55%)	0.29 ^2^
Bacteremia	101 (34%)	9 (41%)	0.49 ^2^
Endocarditis	49 (16%)	2 (9%)	0.37 ^2^
Osteomyelitis	44 (15%)	4 (18%)	0.66 ^2^
Other	33 (11%)	3 (14%)	0.70 ^2^
Epidural abscess	17 (6%)	2 (9%)	0.51 ^2^
Septic arthritis	12 (4%)	2 (9%)	0.26 ^3^
Inpatient consultation performed			
Addiction medicine	164 (55%)	18 (82%)	**0.01** ^2^
Infectious diseases	132 (44%)	11 (50%)	0.58 ^2^
Return to acute care within one year			
Emergency department visit	148 (50%)	15 (68%)	0.10 ^2^
N/A (Death during SA)	5 (2%)	0 (0%)	
Readmission	146 (49%)	13 (59%)	0.39 ^2^
N/A (Death during SA)	5 (2%)	0 (0%)	
MOUD prescription			
Initiated during stay	60 (28%)	5 (31%)	0.79 ^2^
On MOUD prior to admission	87 (29%)	6 (27%)	
Provided on discharge	123 (42%)	6 (27%)	0.18 ^2^
N/A (Death during SA)	5 (2%)	0 (0%)	
PrEP continuum			
HIV screening test performed	168 (56%)	N/A	
Present during admission	0 (0%)	N/A	
Eligible	300 (100%)	0 (0%)	
Documented discussion	2 (1%)	N/A	
Prescribed on discharge	1 (< 1%)	N/A	
Linked to PrEP follow-up care	0 (0%)	N/A	
HIV-specific outcomes			
Viral load collected during admission	N/A	13 (59%)	
Viremia present	N/A	13 (59%)	
Median HIV viral load	N/A	6226 copies/mL	
Median CD4 count	N/A	206 cells/mm^3^	
Antiretroviral therapy POA	N/A	7 (37%)	
Antiretroviral therapy on discharge	N/A	11 (50%)	
Successfully linked to care	N/A	10 (45%)	
Viral load negative within 1 year	N/A	4 (18%)	

*SSTI* Skin and soft tissue infection; *N/A* Not Applicable; *SA* Sentinel Admission; *MOUD* Medications for opioid use disorder, *PrEP* Pre-exposure prophylaxis for HIV

Table statistics reported as Median (Interquartile Range) for continuous factors, and Frequency (Column Percentage %) for categorical factors

Missing data is reported and not included in summary statistics

**Bold** p-value indicates significance at the 0.05 level

^1^Kruskal–Wallis p-value, ^2^Pearson Chi-Square p-value, ^3^Fisher’s Exact p-value

### Outcomes of PWH

Of the 322 patients, 22 had a positive HIV test during admission. Based on chart abtraction, 19 of them had been previously diagnosed with HIV and 3 were newly diagnosed. Among all PWH (n = 22), the median age was 48, with the majority being Black (55%), male (68%), and Medicaid-insured (77%). During admission, 7 of 19 (32%) previously-diagnosed PWH were on antiretroviral therapy (ART; 32%) and 5 (27%) were on medication for opioid use disorder (MOUD). Thirteen of the 22 PWH (59%) had a viral load checked during admission, of whom 100% were viremic with a median viral load of 6226 copies/mL. During hospitalization, 18 PWH (82%) had an infectious diseases consultation. At discharge, 11 PWH (50%) had ART on their discharge medication list, and 14 (64%) had a discharge plan for outpatient HIV follow up. Of the 11 patients with ART on their discharge plan, 4 were on MOUD prior to admission, 1 initiated MOUD during admission, and 2 were prescribed MOUD on discharge. Four PWH were discharged via patient-directed discharge (PDD), while the other 18 had routine discharges. In the year following discharge, 12 PWH (55%) attended at least one outpatient HIV-related visit, 4 (18%) had an undetectable viral load, 15 (68%) returned to the emergency department, and 13 (59%) were readmitted.

### Outcomes of PrEP-eligible patients

Of patients who did not have a positive HIV test reported in the health system prior to sentinel admission (n = 303), 171 (56%) were screened during hospitalization and of those, 168 (98%) were negative. Patients who had negative sentinel admission HIV tests or no positive HIV test in the system were assumed to be PrEP eligible. Among these patients, 203 (68%) were white, 141 (47%) were Medicaid-insured, and 146 (49%) were stably housed. One hundred sixty-four (55%) had an infectious diseases consultation. A total of 231 (77%) had planned discharge, 60 (20%) had PDD, 5 died, 4 had other discharge status. Within one year of discharge, 148 patients (49%) returned to the same emergency department, and almost as many at 146 (49%) were readmitted. Only 2 patients’ charts contained documentation of a PrEP discussion; 1 patient was discharged on PrEP and none had an outpatient visit for PrEP in the year following sentinel admission within the same health system.

## Discussion

In this multisite, retrospective cohort of persons with OUD hospitalized with infectious complications of injection drug use, we found multiple opportunities for improvement across the HIV care continuum. Hospitalizations were lengthy and readmissions were common, highlighting the hospital as a frequent touch point for HIV service delivery in OUD. Among PWH, ART prescribing on discharge along with follow-up care and quality metrics, including viral suppression, were also low. Among HIV-negative PWID, rates of HIV screening were poor (only 56% screened), which is on par with other national studies and likely reflects restrictive consent policies and provider workflow issues [6, 7]. Only one patient was prescribed PrEP on discharge, and there was no evidence of outpatient linkage for PrEP despite a high rate of infectious disesases consultation. Long hospital lengths of stay provided ample time for HIV PrEP counseling, prescription, and discharge planning. Compared to the HIV-negative PWID, PWH had significantly longer lengths of stay, higher rates of unstable housing, and were less likely to be white. They also had lower rates of MOUD use on discharge than the HIV negative cohort.

PrEP uptake among PWID is a known gap in hospital and outpatient settings. Known barriers to PrEP in PWID include patient misconceptions surrounding PrEP and provider bias against PWID [8]. In the acute care setting, limited patient knowledge of PrEP is a well-noted barrier to PrEP initiation, along with limited referral networks for PrEP management [8]. Among outpatient opioid treatment providers, substantial logistical barriers to offering PrEP exist, with only 9.5% of opioid treatment programs in the U.S. reporting capacity to provide PrEP to their clients as of 2019 [9]. Providers cite a variety of concerns including stigma, poor access to PrEP due to insurance restrictions, and a high burden of lab monitoring for patients on PrEP [3]. The lack of adequate follow-up infrastructure may contribute to inpatient providers’ reluctance to prescribe PrEP on discharge, especially in PWID and among providers practicing in non Medicaid-expansion states where PrEP is more difficult to secure.

There were several limitations to our study. First, testing results, care linkage, and follow-up testing could only be evaluated within the same health system as the sentinel admission. Patients may have followed up with care outside of the system; these would have been misclassified as not being linked to care. Second, our data were retrospective and collected entirely via chart review, hindering our ability to discern nuances of scenarios in which clinical decisions may have been appropriate (for example, not screening for HIV in a patient who reports a very recent negative test). Future prospective studies could address these concerns. Additionally, persons for whom no HIV test was collected were assumed to be HIV negative for the purposes of the PrEP eligibility analysis, but some may have in fact been HIV positive and undiagnosed due to absence of HIV testing, a significant assumption that reflects a substantial failure in the HIV care continuum. Finally, people without opioid use disorder were excluded from this study, despite the fact that people who inject stimulants share high risks of HIV acquisition.

Our findings are a call to action for all providers who serve PWID. All clinicians should be aware of the unique HIV risk behaviors and prevention needs for PWID, including the need for comprehensive screening, treatment, and prevention services offered through a status neutral framework [10]. Low-cost, low-barrier interventions like electronic health record-triggered opt out testing, checklists, and order sets are one approach to standardize and increase uptake of evidence-based services for PWID, especially among health systems facing resource limitations. Future studies should explore the ways that information systems could be leveraged to better address this care gap in testing and PrEP provision. Additionally, future research should explore the barriers to ART prescription on discharge given the exceptionally low rates observed here. Hospital-based clinicians, including discharge support staff, should be aware of institutional and community HIV resources. Long-acting injectable options for pre-exposure prophylaxis, while not yet widely available, cost-effective, or FDA approved for PWID, hold promise for engaging these vulnerable patients. Patient-centered educational initiatives on PrEP and HIV prevention may also help improve PrEP uptake and change perceptions on HIV risk. To increase the use of HIV prevention and treatment services in marginalized groups and reach the ambitious aims of the Ending the HIV Epidemic Initiative [2], we need additional research to develop and implement best practices for hospitalized PWID from admission through discharge.

## Data Availability

The datasets used and/or analysed during the current study are available from the corresponding author on reasonable request.

## Abbreviations

ART: Antiretroviral therapy
CHOICE: Continuum of Care in Hospitalized Patients with Opioid Use Disorder;
MOUD: Medications for opioid use disorder
OUD: Opioid use disorder
PDD: Patient-directed Discharge
PrEP: Pre-exposure prophylaxis for HIV
PWH: Persons living with HIV
PWID: People who inject drugs
SA: Sentinel admission
SSTI: Skin and soft tissue infection
